# Shape Tailored Magnetic Nanorings for Intracellular Hyperthermia Cancer Therapy

**DOI:** 10.1038/s41598-017-14633-0

**Published:** 2017-11-01

**Authors:** Carlos S. B. Dias, Talita D. M. Hanchuk, Heberton Wender, Willian T. Shigeyosi, Jörg Kobarg, André L. Rossi, Marcelo N. Tanaka, Mateus B. Cardoso, Flávio Garcia

**Affiliations:** 1 UNICAMP – State University of Campinas, Cidade Universitária Zeferino Vaz, Campinas, CEP 13083-970 Brazil; 2 LNLS – Brazilian Synchrotron Light Source, Rua Giuseppe Máximo Scolfaro, 10000, Campinas, CEP 13083-970, Caixa Postal, 6192 Brazil; 3 LNBio – Brazilian Bioscience National Laboratory, Rua Giuseppe Máximo Scolfaro, 10000, Campinas, CEP 13083-970, Caixa Postal, 6192 Brazil; 4 UFMS – Federal University of Mato Grosso do Sul, Cidade Universitaria, Campo Grande, CEP 79070-900 Brazil; 5 UFSCar – Federal University of São Carlos, Rodovia Washington Luís, Km 235, s/n, São Carlos, CEP 13565-905 Brazil; 6 CBPF – Brazilian Center for Research in Physics, Rua Doutor Xavier Sigaud, 150, Rio de Janeiro, CEP-22290-180 Brazil; 7 LNNano – Brazilian Nanotechnology National Laboratory, Rua Giuseppe Máximo Scolfaro, 10000, Campinas, CEP 13083-970, Caixa Postal, 6192 Brazil

## Abstract

This work explores a new class of vortex/magnetite/iron oxide nanoparticles designed for magnetic hyperthermia applications. These nanoparticles, named Vortex Iron oxide Particles (VIPs), are an alternative to the traditional Superparamagnetic Iron Oxide Nanoparticles (SPIONs), since VIPs present superior heating power while fulfilling the main requirements for biomedical applications (low cytotoxicity and nonremanent state). In addition, the present work demonstrates that the synthesized VIPs also promote an internalization and aggregation of the particles inside the cell, resulting in a highly localized hyperthermia in the presence of an alternating magnetic field. Thereby, we demonstrate a new and efficient magnetic hyperthermia strategy in which a small, but well localized, concentration of VIPs can promote an intracellular hyperthermia process.

## Introduction

Over the last decades, cancer became the second major cause of death in the world^1,2^. Facing this, several therapies were developed and are still under continuous improvement^3–9^. In this scenario, one of the most promising cancer therapies is based on the use of magnetic nanomaterials^10,11^ which can be specially designed for localized hyperthermia^12,13^. This strategy explores the magnetic induction heating through an alternating magnetic field together with the fact that tumor cells are more heat sensitive than healthy cells^14^. This approach created a demand for the development of highly specialized nanoparticles (NPs), which could be selectively absorbed by the tumor tissue and generate the targeted heating. Currently, most of the NPs used for magnetic hyperthermia are small iron oxide (magnetite or maghemite) spheres with sizes smaller than 15 nm, which are functionalized with organic compounds such as PEG or Dextran to reduce their cytoxicity^15^. These NPs are commonly called Superparamagnetic Iron Oxides Nanoparticles or SPIONs^16^ and have already been tested in clinical trials^17^ for various cancer types, such as brain and prostate cancers^7,18–20^. Recently, MagForce® Nanotechnologies AG^21^, a company from Germany, received the European approval to use SPIONs for human magnetic hyperthermia cancer therapy.

Until recently, the concept of a superparamagnetic NPs has been the backbone on the development of magnetic hyperthermia^12^. It has been widely optimized in terms of size, saturation magnetization and magnetic anisotropy^22–24^, aiming at the highest response under usual conditions of applied magnetic field (amplitude and frequency). Nonetheless, the work of Liu *et al*.^25^ introduced a new class of iron oxide based nanoparticles presenting an improved magnetic hyperthermia response, when compared to standard SPIONs, while preserving low cytotoxicity. It was demonstrated that these characteristics are due to the unusual shape and size of these particles, which explore a peculiar configuration known as magnetic vortex^26^. The peculiarity of such magnetic states comes from their distinct magnetic moment configuration. In this case, the magnetic moments from the iron oxide curls in concentric circles, confining the magnetic flux within particle, creating hence a particle with no magnetic pole, a micromagnetic ouroboros.

In the present work, we have synthesized a new class of nanoparticles named as Vortex Iron oxide Particle, or simply VIPs, with distinct dimensions in the nanometer range, while their magnetic dynamics and vortex states were characterized through a combination of micromagnetic simulations and magnetization measurements. *In vitro* experiments demonstrated that the synthesized nanoparticles were not cytotoxic and could further be used to evaluate their magnetic hyperthermia in the presence of mammalian cells. Magnetic hyperthermia experiments showed evidence of a very localized response, promoting cell death with no environmental heating. Finally, images from Confocal microscopy and Scanning Transmission Electron Microscopy (STEM) showed that the VIPs had been internalized by the cells. The internalization phenomena of the VIP resulted in intracellular hyperthermia, allowing us to conclude that the VIP when compared to traditional SPION would be a much more localized and efficient particle for magnetic hyperthermia.

## Results and Discussion

**Figure 1:**
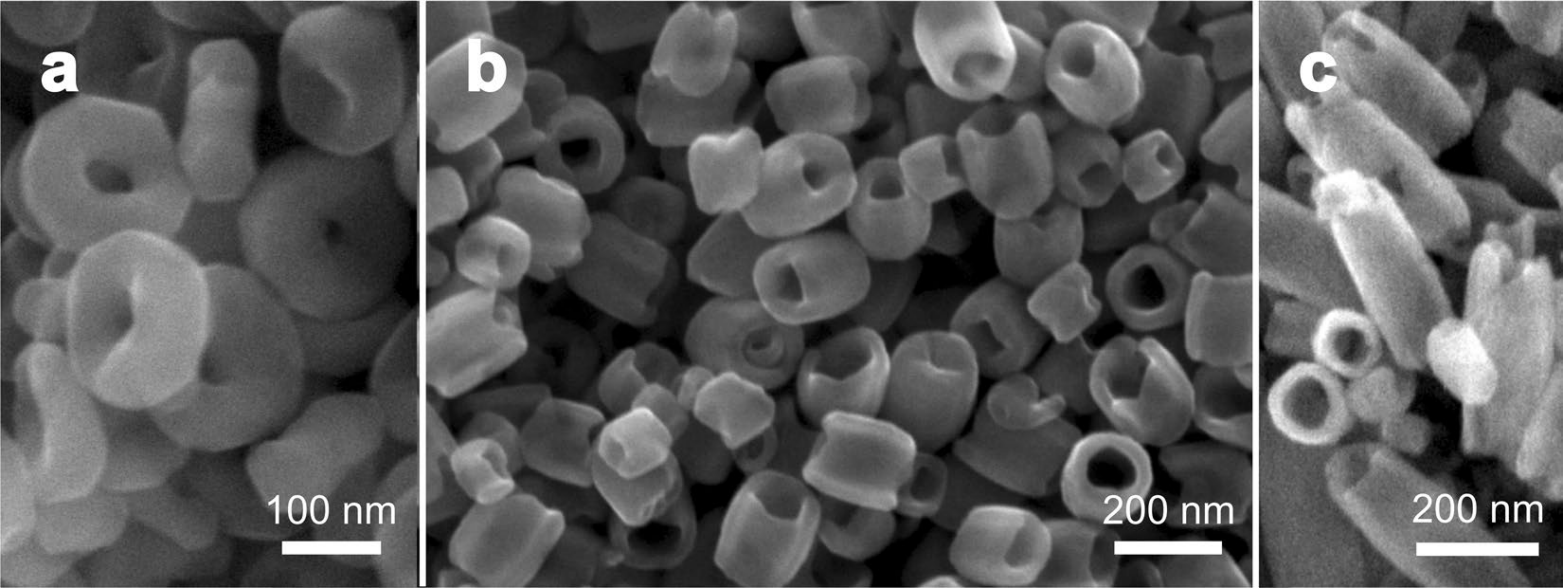
SEM image of iron oxide nanoparticles prepared by hydrothermal reaction: (**a**) VIP1 (nanoring-like particles); (**b**) VIP3 (aspect-ratio close to 1); and (**c**) VIP6 (nanotube-like particle).

Three distinct sets of Vortex Iron-oxide Particles, hereafter denominated VIP1, VIP3 and VIP6, were synthesized by using a previously reported methodology^27,28^. The VIPs morphology was investigated by field emission scanning electron microscopy (FESEM) and the particle size distribution was obtained by measuring the height, internal and external diameter of at least 400 particles. Figure 1 presents a collection of SEM images of three distinct samples while the histograms for VIP1, VIP3 and VIP6 are provided in Figure S1 (Supporting Information).

These three samples were synthesized in order to investigate the influence of the aspect ratio (here defined as the VIP’s height divided by its external diameter) on the magnetic, physicochemical and biological properties of the samples. X-ray diffraction (XRD) and X-ray absorption near edge spectroscopy (XANES) experiments were performed to probe the crystallographic structure and chemical composition along the VIP synthesis. These results are presented in the Supporting Information (Figures S2 and S3) and indicate that the obtained VIPs are mostly composed of magnetite. Magnetic characterization was performed at different temperatures and all samples were measured in the dried state. Table 1 presents the saturation magnetization derived from Figure S4. It is possible to observe that the process of NPs reduction was not equally efficient for all nanoparticles. Nevertheless, the saturation magnetization results corroborate that the VIPs are mostly composed of magnetite. The hyperthermia measurements were performed and the Specific Absorption Ratio (*SAR*) and the Intrinsic Loss Power (*ILP*) were determined; both are commonly used to quantify the efficiency of a magnetic hyperthermia system. Table 1 summarizes the *SAR* and the *ILP* calculated for the three studied particles, together with their respective magnetization of saturation (M_s_) for comparison. VIP1 is the one presenting the highest magnetic response (*SAR* = 426 Wg^−1^, ILP = 5.6 nHm^2^kg^−1^) and the largest saturation magnetization (89 emu g^−1^). The fifth and sixth columns show the normalized *SAR* and *ILP* by the M_s_ indicating that the differences on the magnetic hyperthermia are mainly due to the Saturation Magnetization and that the samples are consistent.

**Table 1:** *SAR* values calculated for 200 Oe applied field together with the normalized ILP values. Saturation Magnetizations are also presented for comparison.

Nanoparticle	*SAR* at 200 Oe (Wg^−1^)*	*ILP* (nHm^2^kg^−1^)	Saturation Magnetization (emu g^−1^)	Normalized *SAR* (W/emu)	Normalized *ILP (p*Hm^2^/emu)
VIP1	426	5.6	89	4.7	1.2
VIP3	368	4.8	79	4.6	1.0
VIP6	401	5.3	81	4.9	1.1

^*^For a frequency of 300 kHz.

In addition, a series of micromagnetic simulations were made to confirm the existence of magnetic vortices within the three morphologically (size and shape) distinct VIPs. Figure S5 presents a representative image of the simulated nanoparticle where NP height (H), NP outer diameter (ExtD) and NP inner diameters (IntD) are presented.

**Figure 2:**
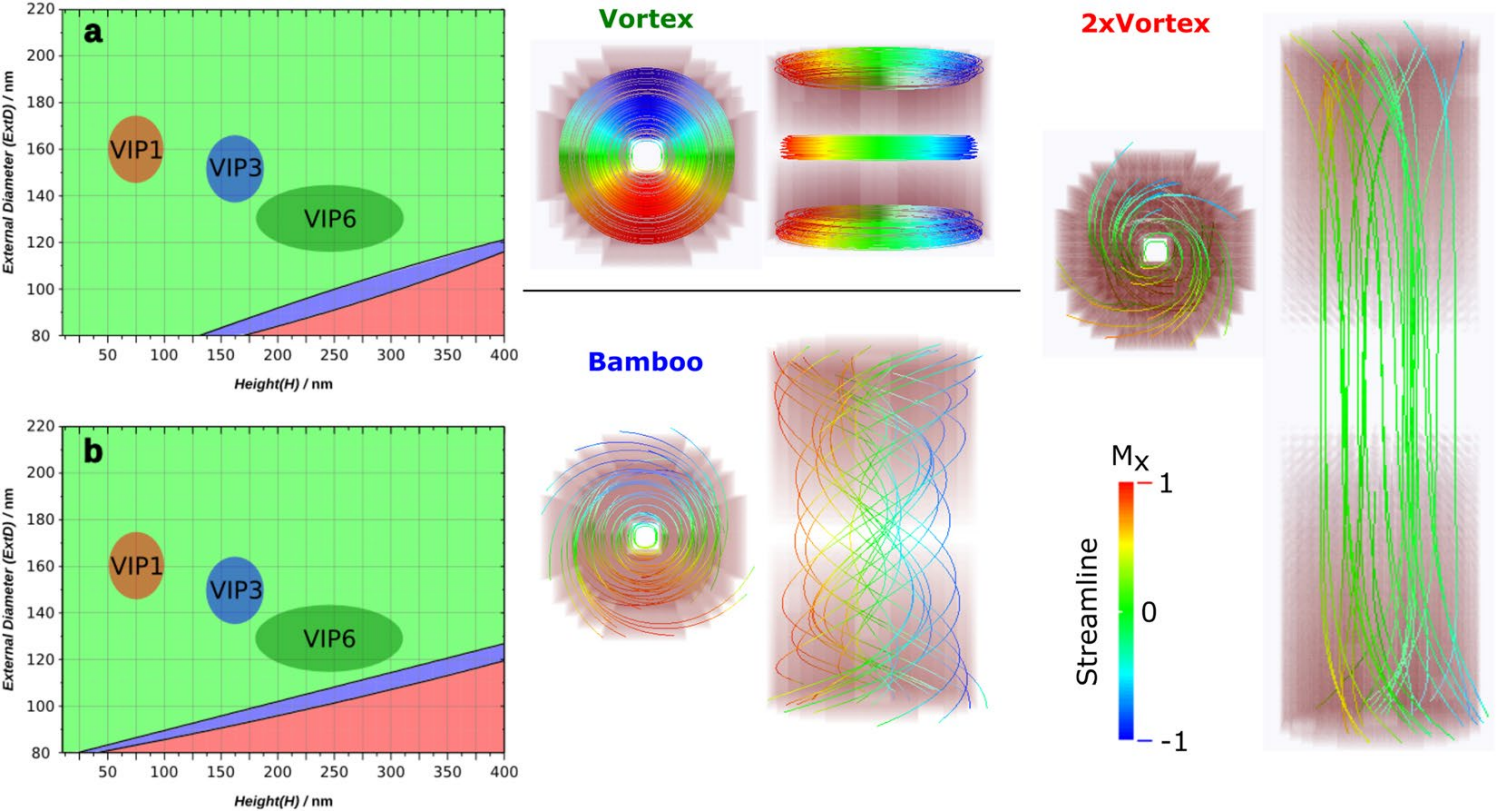
Sections of the 3-D phase diagrams for a fixed internal diameter of (**a**) 50 nm, and (**b**) 70 nm. Vortex (green), Bamboo (blue) and 2xVortex (red) states are presented. The three regions identified as VIP1, VIP3 and VIP6 are the morphologic position within the phase diagram of the respective nanoparticles with respect to their standard deviations. The rest of the phase diagram, has been omitted for space restriction.

Thus, sweeping across different series of (H, ExtD, IntD) trios, the results were used to compose a 3-D micromagnetic phase diagram. In Fig. 2 two sections from the tridimensional diagram at IntD = 50 nm and IntD = 70 nm while ExtD and H varied from 80 to 210 nm and from 5 to 400 nm, respectively, show the micromagnetic simulations of the synthetized VIPs. Together, the magnetic states depicted on the phase diagram as green, blue and red colors represent the Vortex, Bamboo and 2xVortex states illustrated with the streamlines following the magnetic flux within the particle.

Also, in Fig. 2, the regions highlighted as VIP1, VIP3 and VIP6 representing the size distributions within the micromagnetic phase diagram with respect to their standard deviations show that the synthesized NPs are within the magnetic vortex region, indicating that these particles can indeed be classified as Vortex Iron oxide Particles. Furthermore, it is worth noting that the observed magnetic states are in agreement with similar nanoparticles and their micromagnetic phase diagram^27,29^, at the same time, part of the presented micromagnetic phase diagram are new and also represent an unpublished contribution to the current knowledge regarding the VIP shape and micromagnetic state. Finally, we must highlight that although the synthesized particles respect the necessary dimensions to present a vortex state, the small remanence measured from Figure S4 should be interpreted as an effect of the polydispersity of the sample, which was not considered in the magnetic hysteresis simulation.

Following, the cytotoxicity of these particles was evaluated for their possible application as a bioengineered material^30–33^. Human Embryonic Kidney (HEK293) cells were exposed to different concentrations of the VIPs for 24 and 48 h (Figure S7), and no cytotoxic effect was observed.

**Figure 3:**
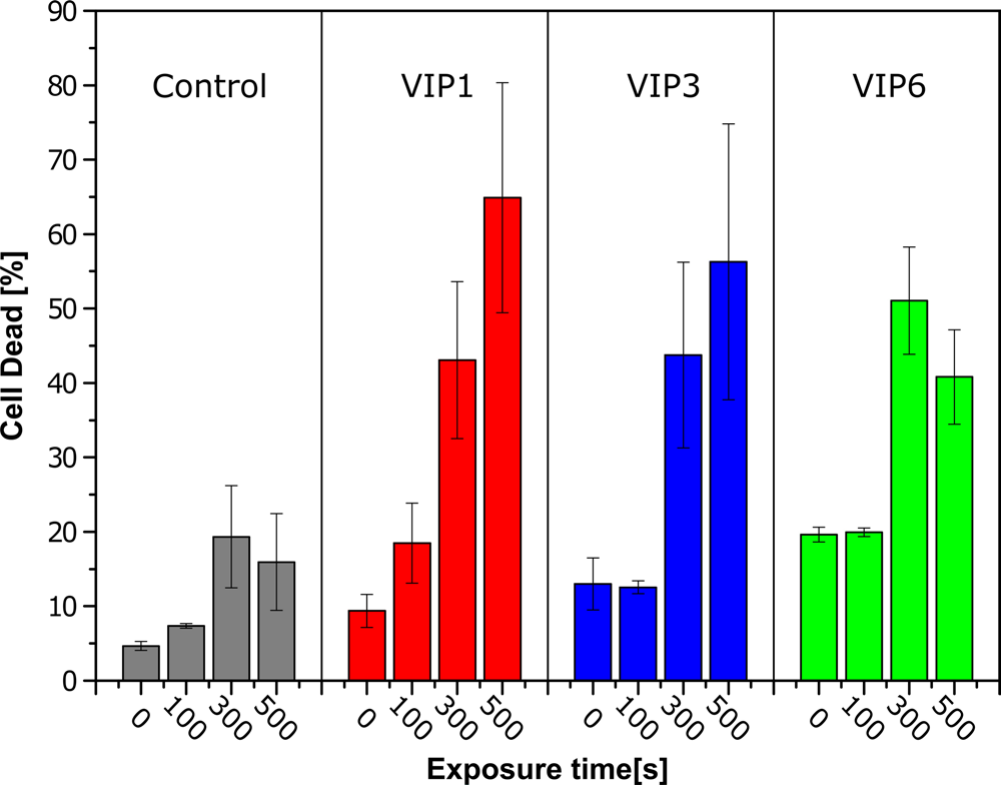
Effects of *in vitro* hyperthermia experiments on cell death rates after nanoparticle treatments. HEK293 cells were treated with indicated nanoparticles (24 h of incubation in the presence of 100 µg ml^−1^ of nanoparticles). The control cells are shown in gray and VIP1, VIP3 and VIP6 nanoparticles treated cells are shown in red, blue and green, respectively. Cell death was assessed by Flow Cytometry using determination of dead cells by propidium iodide incorporation.

Further *in vitro* experiments with mammalian cells were performed to evaluate the potential of these nanoparticles for hyperthermia applications. This experiment was performed on HEK293T previously incubated with VIPs for 24 h. After being exposed to a magnetic field, cell viability was measured by Flow Cytometry (FC) through cell staining with propidium iodide, which quantifies the number of non-viable cells. Figure 3 summarizes these results. Different colors were used to distinguish between the control group and the three distinct VIPs.

Firstly, it is important to point out that the *in vitro* hyperthermia experiment has been carried outside of the cell culture environment. Consequently, it was not possible to guarantee a stress-free environment for the cells resulting in significant cell stress for longer treatment times. Besides, even working below the accepted biologic limits, there are no guarantees that the magnetic field does not affect the cells. The consequences of these effects are observed with the control group (gray) shown in Fig. 3. Another important point to be highlighted is the absence of observable sample heating correlated with an alternating magnetic field exposure during the experiment. This was somehow expected, since the NPs concentration was considerably low compared to the culture medium in which the cells were initially incubated with the VIPs. In a typical experiment, the SAR measurements presented in Table 1 were performed using 1 mg/mL VIP aqueous suspensions. On the other hand, for the *in vitro* experiments, the cells were initially incubated with 0.1 mg/mL VIP suspensions and then washed in order to remove any free non-interactive VIP. This procedure resulted in a concentration at least 10 times smaller in the *in vitro* experiment, which is too small for generating an observable environmental heating.

Therefore, it is clear that the VIPs in the presence of magnetic field induced cell death pointing a common behavior for all three nanoparticles. Firstly, we can observe that most of the inflicted damage in the cells which leads to the cell death occurs after 100 s of applied magnetic ac field. This is supported by the comparison between VIP groups and the control one. On the other hand, the number of dead cells is not significantly different when 300 and 500 s are compared.

**Figure 4:**
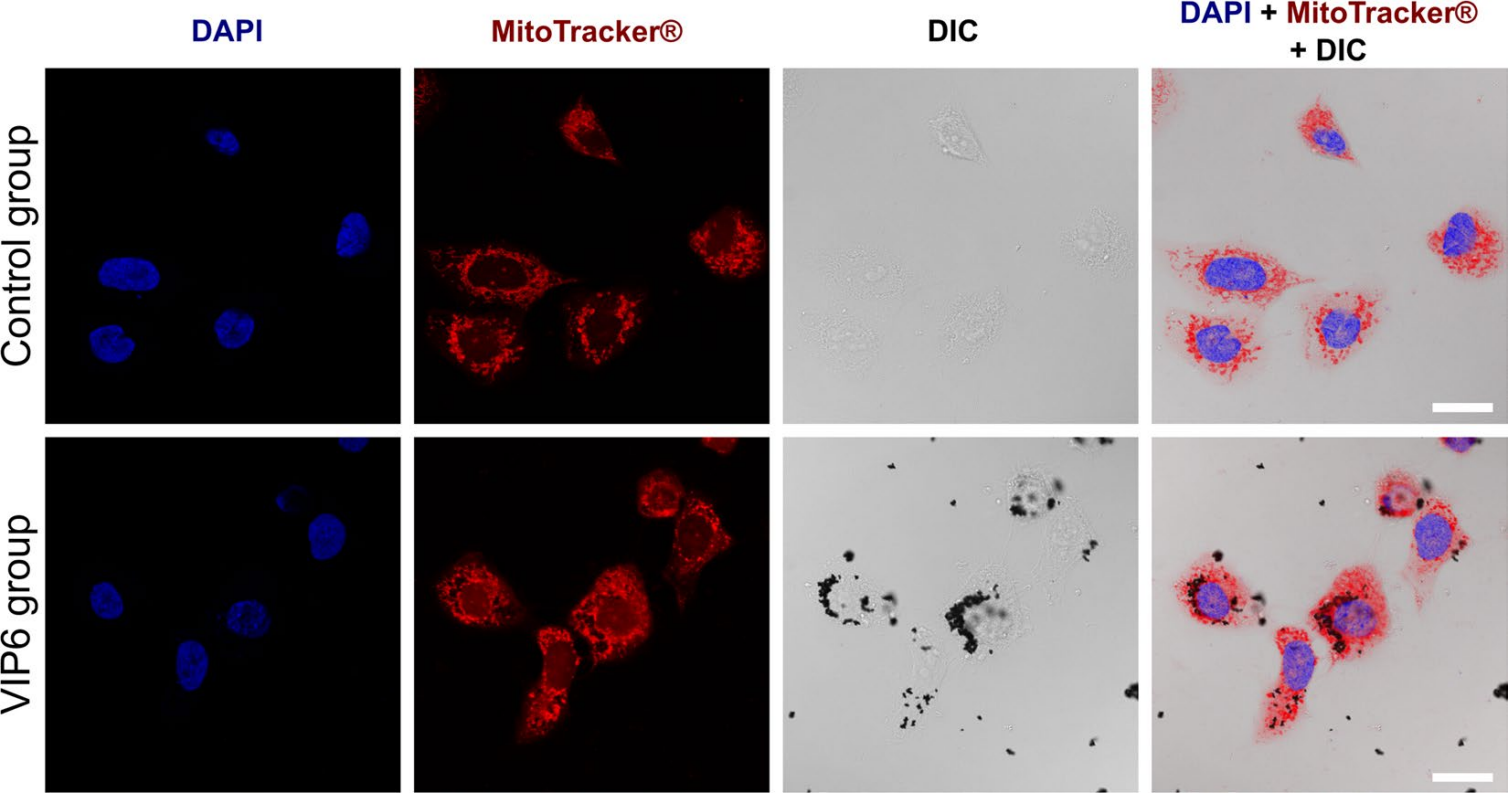
Confocal images of U2-OS fixed control cells and cells treated for 24 h with VIP6. Fluorescent signals of Hoechst-nuclei marker (blue) and Mitotracker**®**-mitochondria marker (red) indicate nucleus position and cell boundaries, respectively. The DIC image shows VIP6 agglomerations. Combination of all fluorescent and DIC images indicates VIP6 localization within the cells. Scale bar is 20 µm.

Figure 4 shows, by confocal microscopy, the internalization of VIP6 after 24 h of treatment as a tentative to better understand the biological effect associated to these particles. Nuclei of U2-OS cells were stained in blue with Hoechst while mitochondria were labeled in red with Mitotracker**®** deep red. The mitochondria labeling was used as an indicative of cell boundaries. Differential Interference Contrast (DIC) allowed the visualization of magnetite nanorings agglomerates due to the low light transmission property of the material resulting on dark contrast regions within the DIC image. Ultimately, the superposition of all images of the sample group allows the observation that the nanoparticles are mainly located close to the nuclei. Similarly, conventional optical images were taken for HEK293T cells after the treatment with all three nanoparticles of interest. The HEK293T results are not shown since the cytoplasm of these cells are considerably smaller than U2-OS and, consequently, NPs location is less evident.

**Figure 5:**
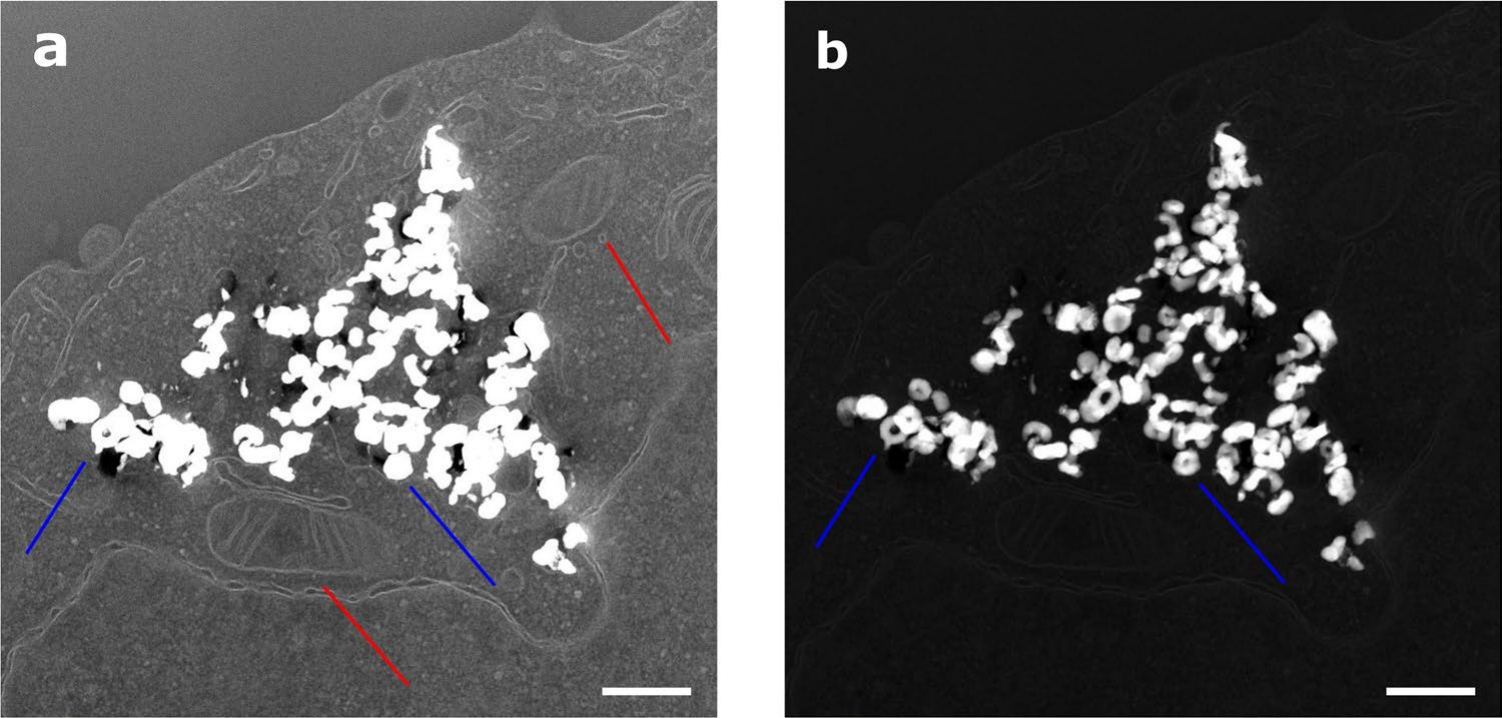
STEM dark field images of VIP1 treated HEK cells ultrathin section. The HEK cells were treated following the same procedures as if would be expose to the magnetic field. (**a**) STEM ADF imaging for detailing cell mitochondria (red arrows), but saturated VIP with indistinctive ring-shape structure (blue arrows); (**b**) STEM HAADF for detailing VIP1 structure with distinctive ring-shape structure (blue arrow). Scale bar is 500 nm. (Additional micrographs are also shown on Figures S8 and S9).

Since individual nanoparticles are too small to be seen using an optical microscope, we can only assume that the dark spots observed in the DIC images correspond to nanoparticle agglomerates inside the cells. Due to the fact that the presented confocal technique is not able to discriminate between agglomerates, creating some uncertainty on whether the nanoparticles are inside or on the surface of the treated cells, HEK293T cells were fixed, dehydrated, embedded in resin, sliced using an ultramicrotome and further imaged by STEM (Fig. 5).

The micrographs shown in Fig. 5 were obtained in scanning mode (STEM) using low and high angular annular dark field (ADF and HAADF, respectively) detection modes. For a better cells contrast, low angle collection was selected using ADF. Afterward, keeping the same field of view, HAADF was selected increasing the nanoparticle contrast. All observations were performed using minimal dose to prevent sample radiation damage. These images allowed the observation of individual nanoparticles within the cell structure, confirming that the cells internalized the VIPs. Finally, we can argue that the distinctive flow cytometry backscattering signals observed (Figure S10) for all VIP treated cells are correlated to the presence of internalized VIP within the cell. Therefore, STEM images together with the flow cytometry measurements strongly suggest that the VIPs are internalized and working in favor of an intracellular hyperthermia effect.

A modified MILC protocol presented by Galimard *et al*.^34^ was employed to estimate the nanoparticle mass concentration inside the cells. Briefly, after incubation, the cells were washed with phosphate buffered saline (PBS) to remove the non-internalized nanoparticles. Following, the cells were trypsinized and the number of cells were estimated by cell counting. Cells were centrifuged and re-suspended in hydrochloric acid, resulting in a yellow color solution which was analyzed by UV–Vis spectroscopy and compared to the references. The amount of iron absorbed by the cells was used to estimate the nanoparticle concentration per volume (Table 2).

**Table 2:** Intracelular VIP mass concentration obtained from the MILC protocol.

Nanoparticle Name	m_FE_ (pg/cell)	M_FE_ (mg/ml)*
VIP1	106 ± 19	11 ± 2
VIP3	80 ± 15	8 ± 2
VIP6	81 ± 16	8 ± 2
*in vitro* incubation	—	0.1

m_FE_ is the mass of iron oxide divided by the number of cells and M_FE_ is the concentration of iron oxide inside a mammalian cell, considering an average cell volume of 10 pL^36^. Also, the iron oxide concentration used during the nanoparticles *in vitro* incubation for the hyperthermia experiments is presented for comparison. *Estimated value for a mammalian cell with 10 pL volume^36^.

As one can observe, the presence of the VIP inside the HEK293T cells are in a concentration of at least 80 pg/cell. Thus, the observed cell death is a consequence of intracellular hyperthermia process. In this scenario, only the cells with internalized VIPs are affected by the magnetic field and the majority of affected cells are killed up to 300s. After 300 s, all nanoparticles presented a similar cell viability result. This behavior corroborates the proposed scenario of the VIP concentration being insufficient to generate environmental heating, as well as keeping the heat localized within the cells. Therefore, this behavior would be two times more effective because it would only affect cells with internalized VIPs and save VIP free cells from death induced by hyperthermia.

Taking the VIPs dimension (as large as 100 nm) into consideration, we believe that the internalization is driven through active mechanisms, such as phagocytosis or endocytosis, since for this size range the osmotic mechanism can be excluded. However, the internalization mechanism determination is beyond of the scope of this work. Individually, VIP1 nanoparticles presented the best performance when compared to the other two types of particles studied. It presented larger SAR values (Table 1) in addition to more stable vortex state, shown by the micromagnetic simulations. Besides, the *in vitro* hyperthermia experiment of VIP1 had the most consistent results, with a tendency of promoting cell death in shorter exposure periods (100s).

## Conclusion

In the present paper, we demonstrated that mammalian cells have a tendency to internalize the synthetized VIPs with no significant cytotoxicity effect. Furthermore, the observed concentration of the VIP inside the cells promoted a much more efficient path to cell death through magnetic hyperthermia. We observed that most of the VIPs were found inside the cells, and that this internalization was similar for all the tested particles independently of differences on their aspect ratio. This result indicates no significant selectivity of synthesized VIPs, demonstrating that future designs may benefit of a broader range concerning the VIPs aspect ratio. After quantifying the concentration within the cells, we noticed that it was at least 80 times larger than the overall concentration used for the entire experiment. Due to this high intracellular concentration, the magnetic field was able to promote a localized heating, which resulted in a much more efficient path to cell death, at the same time that the overall concentration was unable to promote environmental heating. Such a process was named intracellular hyperthermia, that, to our knowledge, is an unusual phenomenon, despite its significance in the development of nanoparticles for magnetic hyperthermia. It is common to most hyperthermia procedures, to ignore any intracellular hyperthermia phenomena, since the majority of the works in this field focus on environmental heating, targeting entire tissues with no selectivity at the cellular level. These procedures target tissues and cells indiscriminately denying the benefits of a more selective targeting at cellular levels. As a result, we believe the intracellular hyperthermia presented in this work is the natural next step for magnetic hyperthermia. The presented strategy would have a better selectivity and a much higher efficiency, at the same time the synthesized nanoparticles would also provide new forms of drug delivery and intracellular targeting to be further explored on the future.

## Experimental Methods

### Materials

Iron (iii) chloride (FeCl_3_), monosodium phosphate (NaH_2_PO_4_. H_2_O) and sodium sulfate (Na_2_SO_4_) were purchased from Sigma-Aldrich. All chemicals were used without further purification. The H_2_/He (5%) gas mixture was prepared using H_2_ (99.99%) and He (99.99%) by mass controllers with set points of 2.5 mL min^−1^ and 47.5 mL min^−1^, respectively.

### Synthesis of the Iron Oxide Nanorings

The iron oxide particles were synthesized by two steps process. First, hematite (α-Fe_2_O_3_) nanorings were prepared following the procedure reported by Jia *et al*.^27,28^. In a typical experiment (for sample VIP1), a solution of FeCl_3_ (0.02 m), NaH_2_PO_4_ (0.1 mm) and Na_2_SO_4_ (0.55 mm) was mixed at room temperature. Different concentrations of NaH_2_PO_4_ were tested in order to investigate their influence over the NPs morphologies. The samples were named as VIPβ for a respective NaH_2_PO_4_ concentration of β10^–4^ m. In all cases, a total volume solution of 80 mL was transferred to a Teflon-lined autoclave reactor (100 mL) that was closed and maintained at 220 °C for 48 h. After cooling down to room temperature, a red powder was precipitated by centrifugation and dried at 80 °C under vacuum conditions, corresponding to α-Fe_2_O_3_ nanoparticles (hematite). The second step consisted in reducing the as-cast hematite nanoparticles in order to convert them to magnetite. The system was then annealed for 1 h at 420 °C in an H_2_/He (5%) 50 mL min^−1^ flux atmosphere.

### Characterization

The morphology of the samples was investigated in a FEI Inspect F50 field emission scanning electron microscope (FESEM, 1.2 nm resolution, operated at 10–30 kV, secondary electrons detector, at the Brazilian Nanotechnology Laboratory). All samples were prepared by deposition of dried nanoparticles on a carbon tape on top of a Si (100) substrate. The corresponding particle size distribution was obtained by measuring the height as well as internal and external diameters of more than 400 particles duly aligned to the main electron beam axis.

### Hyperthermia Measurements

The experimental *SAR* determination was done using an inducing heating system (Ambrell, Easy Heat 4.2 kW) with eight round water cooled coil operating at an alternating magnetic field of 300 kHz and amplitude up to 40 kA m^−1^. A known volume (2 mL) of suspended magnetic nanoparticles in water (1 mg mL^−1^) was placed into a cylindrical glass vial, which was thermally isolated, preventing any heating arising from the coil while mitigating the losses to the environment. Measurements were done along 180 s in which the temperature was probed by an optic fiber thermometer (Optocon, DE) at every 1 s with a resolution of 0.1 °C. The *SAR* results were obtained from the slope of the curve following the expression shown in Equation 1.

### *In vitro* hyperthermia

*In vitro* hyperthermia was performed on individual wells containing about 5 × 10^4^ HEK293T cells in DMEM supplemented with serum and penicillin/streptomycin and maintained at 37 °C in a 5% CO_2_ atmosphere for 24 h. Then, nanoparticles suspended in complete medium (100 µg mL^−1^) were added to each well, which were incubated at 37 °C in a 5% CO_2_ atmosphere for 24 h. It is worth to highlight that the concentration used during *in vitro* hyperthermia experiments is 10 times lower than those used on NP’s *SAR* characterization, i.e., without cells. After incubation, each well was washed with DMEM in order to remove any free nanoparticles and leaving only those that actually interacted with the cells. Each well was then exposed to the alternating magnetic field of 200 Oe and 300 kHz for different times (0, 100, 300, 500 seconds). Finally, cells were evaluated using flow cytometry with propidium iodide as label for dead cells. All measurements were done on triplicate.

### Flow cytometry

Flow cytometry was carried out in a Flow Cytometry FACSCanto II - BD Biosciences. After magnetic field exposure, the cells were trypsinized and stained with Propidium Iodide for 15 minutes at room temperature in the dark. Following, the cells where analyzed by the flow cytometry where PI positive cells were considered dead cells and PI negative were considered viable cells. All measurements were composed of at least 15000 events.

### Confocal optical microscopy

Confocal microscopy was performed using U2-OS cells. Cells were seeded over a microscope cover slip into 24 cell culture wells at 5 × 10^4^ cells per well in DMEM and maintained at 37 °C in a 5% CO_2_ atmosphere for 24 h. Then, the cells were incubated with suspended VIP1, VIP3 or VIP6 (100 µg mL^−1^) for 24 h at 37 °C in a 5% CO_2_ atmosphere. Untreated wells were used as control. Thereafter, mitochondria were stained with Mitotracker**®** Deep Red (Life Technologies) and then cells fixed with paraformaldehyde and the nuclei stained with Hoechst. Finally, microscope slides were taken to a Leica SP-8 confocal microscope for imaging (LNBio-CNPEM).

### TEM sample preparation

Following the same procedure described above, HEK cells were incubated for 24 h in the presence of the nanoparticles. After the cell cultures were gently washed with medium to remove free nanoparticles. Following the adherent cells were removed from the well by scraping and fixed in 2.5% glutaraldehyde and 4% paraformaldehyde in 0.1 M sodium cacodylate buffer (pH 7.4). The samples were rinsed several times with cacodylate and then treated with 1% osmium tetroxide and 1% potassium ferrocyanide in the same buffer. The samples were rinsed and dehydrated in an ethanol series (30 to 100%), embedded in epoxy resin and then polymerized at 60 °C for 48 h. Ultrathin sections < 150 nm were obtained in an RMC ultramicrotome.

### STEM Images

Scanning transmission electron microscopy (STEM) was performed on a JEOL 2100 F instrument operated at an accelerating voltage of 200 kV with a 0.7 nm spot size. STEM dark field images were obtained using a JEOL low or high angular annular dark field (ADF and HAADF, respectively) detector.

### MILC Protocol

Iron taken by the HEK cells were quantified using the MILC protoco^34^. After incubation the well was washed with phosphate buffered saline (PBS) in order to remove any free nanoparticles and leaving only those that actually interacted with the cell. Following, the cells were trypsinized and re-suspended in DMEM. Aliquots were taken for numbering using a Countess® II FL Automated Cell Counter and the rest centrifuged to get a pellet. Finally, the pellets were dissolved in hydrochloric acid (35%) overnight ending with the yellow color characteristic of tetrachloroferrate ions FeCl_4_^−^. The tetrachloroferrate solution UV–Vis absorption spectra were taken on an 8453 Agilent Ultraviolet-Visible Spectrometer and the amount of iron was defined with the help of a reference solution. All measurements were done on triplicate

### Micromagnetic simulations

The micromagnetic simulations were performed using the OOMMF package^35^. This package provides the total energy of a simulated magnetite nanoparticle as the sum of independent energy terms: the exchange energy, the self-magnetostatic energy, the surface anisotropy energy, and the Zeeman energy. The magnetite magnetic parameters used for the simulations were the following: saturation magnetization Ms = 485 kA m^−1^; exchange stiffness A = 13.2 pJ m^−1^; magneto crystalline anisotropy constant K1 = −136 µJ m^−3^; cubic anisotropy with z axis defined along the axis of symmetry of the cylinder; and damping factor α = 0.5 (chosen based on a reasonable time to reach the equilibrium magnetization state). The system was discretized in cubic cells of 5 × 5 × 5 nm^3^, in agreement with the magnetite exchange length (4.9 nm).

### Supporting Information

Supporting information includes size distribution for the height, internal diameter and external diameters of the synthesized nanoparticles (VIP1, VIP3 and VIP6); X-ray diffraction patterns of VIP3; X-ray absorption near edge spectroscopy of VIP3; magnetization measurement of synthesized VIPs; representative images of a simulated nanoparticle; simulated magnetic hysteresis for a monodisperse ensemble of randomly oriented VIPs; representative image of simulated magnetic phases; *in vitro* cytotoxicity assay after 24 and 48 hours of incubation in the presence of VIPs, and representative flow cytometry measurement. This material are available in the online version of the paper.

## Electronic supplementary material

Supplementary Information
