# Supplementary material for: Shape Tailored Magnetic Nanorings for Intracellular Hyperthermia Cancer Therapy
Source: Sci Rep. 2017 Nov 1;7:14843. doi: 10.1038/s41598-017-14633-0 (PMC5665982; doi:10.1038/s41598-017-14633-0)
Supplement: Supplementary file 1 — Supplementary Information [file 41598_2017_14633_MOESM1_ESM.pdf]

Supporting Information

Shape Tailored Magnetic Nanorings for Intracellular Hyperthermia Cancer Therapy

Carlos S. B. Dias[1,2], Talita D. M. Hanchuk[1,3], Heberton Wender[4], Willian T. Shigeyosi[2,5], Jörg Kobarg[1], André L. Rossi[6], Marcelo N. Tanaka[6], Mateus B. Cardoso[2,7], and Flávio Garcia[6]**

1 - UNICAMP – State University of Campinas, Cidade Universitária Zeferino Vaz, Campinas, CEP 13083-970, Brazil

2 - LNLS – Brazilian Synchrotron Light Source, Rua Giuseppe Máximo Scolfaro, 10000, Campinas, CEP 13083-970, Caixa Postal 6192, Brazil

3 - LNBio – Brazilian Bioscience National Laboratory, Rua Giuseppe Máximo Scolfaro, 10000, Campinas, CEP 13083-970, Caixa Postal 6192, Brazil

4 - UFMS – Federal University of Mato Grosso do Sul, Cidade Universitaria, Campo Grande, CEP 79070-900, Brazil

5 - UFSCar – Federal University of São Carlos, Rodovia Washington Luís, Km 235, s/n, São Carlos, CEP 13565-905, Brazil

6 - CBPF – Brazilian Center for Research in Physics, Rua Doutor Xavier Sigaud, 150 Rio de Janeiro, CEP - 22290-180, Brazil

7 - LNNano – Brazilian Nanotechnology National Laboratory, Rua Giuseppe Máximo Scolfaro, 10000, Campinas, CEP 13083-970, Caixa Postal 6192, Brazil

Address correspondence to

Mateus B. Cardoso

cardosomb@lnnano.cnpem.br

Flávio Garcia

fgarcia@cbpf.br

The concept of superparamagnetic NPs is the backbone on the development of magnetic hyperthermia. The energy lost in the form of heat by a ferrofluid system (supermagnetic particles dispersed on a fluid) can be interpreted as the contribution of three mechanisms: susceptibility losses, hysteretic heating and stirring[29].

The stirring mechanism is associated to the NP dragging through the fluid due to the magnetic field gradient, dipolar inter-particle interactions or any other driving force. In any case, this mechanism can be neglected, since it is very dependent on the environment and NP confinement. During an *in vivo* application, it is possible to assume that NPs are likely to be confined in a tissue or attached to the cell membrane[27,30]. Thus, NP's stirring is largely limited and can be disregard as a heating mechanism.

Conventional superparamagnetic systems, such as the SPIONS, present a heating mechanism known as susceptibility loss. This mechanism is characterized by two relaxations times, namely, the Néel (τ_N) and Brown relaxations (τ_B) which describe the loss of the remanent magnetization[31] found on superparamagnetic systems. Nevertheless, there is a critical particle size, considerably larger than the typical SPION size (> 20 nm), in which the NP magnetization will not relax ($\tau_{N,B} \rightarrow \mathbb{N}$) and the NP is so called blocked. Beyond this critical size, the third heating process, i.e. the hysteretic heating, is the dominant effect and no susceptibility loss is observed.

Finally, the hysteretic heating is conceptually more efficient than the susceptibility loss, but, in most cases, a blocked NP will present a large remanent magnetization, resulting on an enhanced dipolar interaction and a large probability of NP agglomeration/aggregation. Alternatively, in this work introduces the Vortex Iron oxide Particle (VIP) presenting an enhanced hysteretic response while keeping a low remanent magnetization. These characteristics arise from its peculiar shape and intermediated size which explores a magnetic configuration known as magnetic vortex[32]

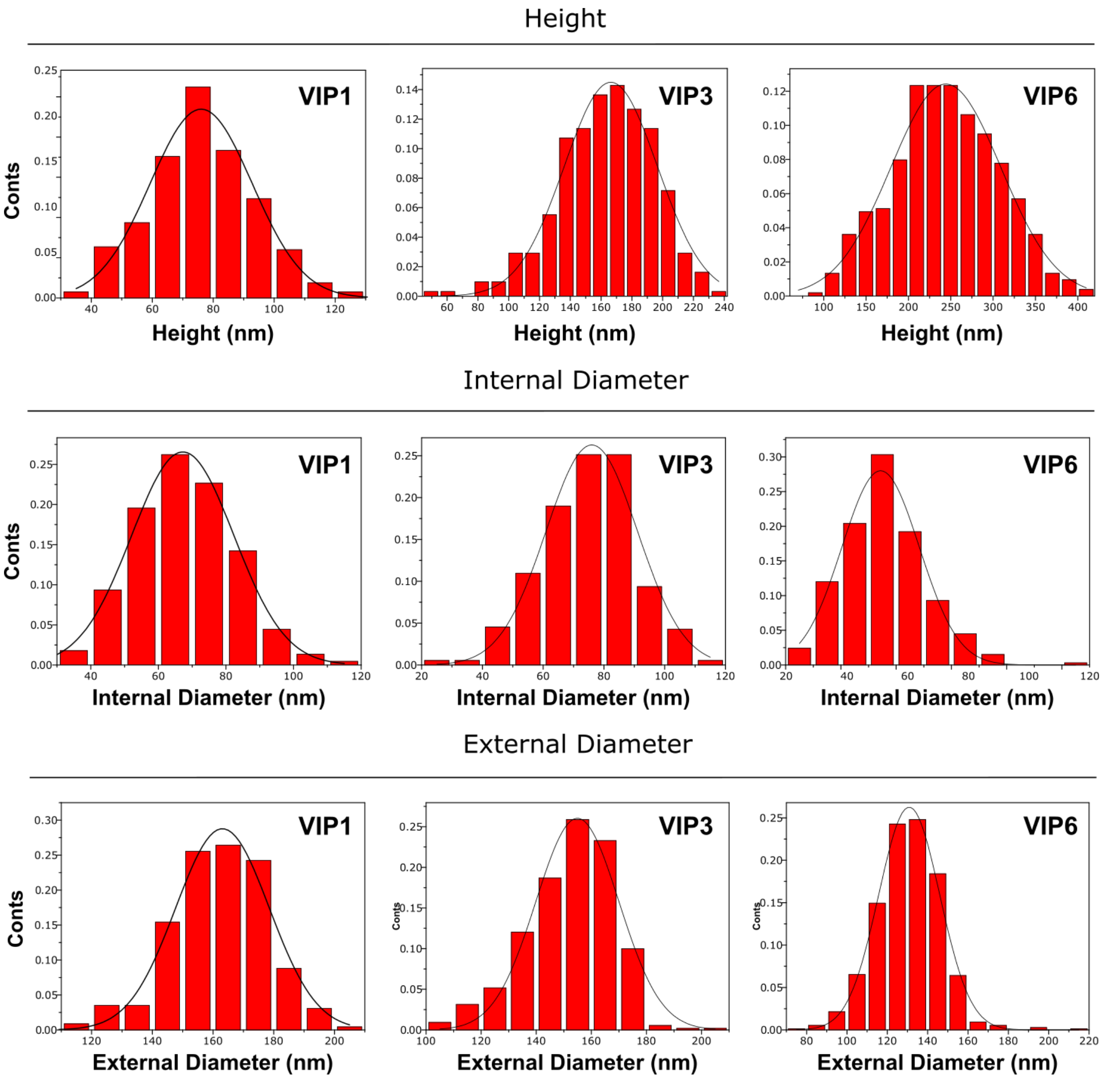

Figure S1. Histograms for VIP1, VIP3 and VIP6 morphology.

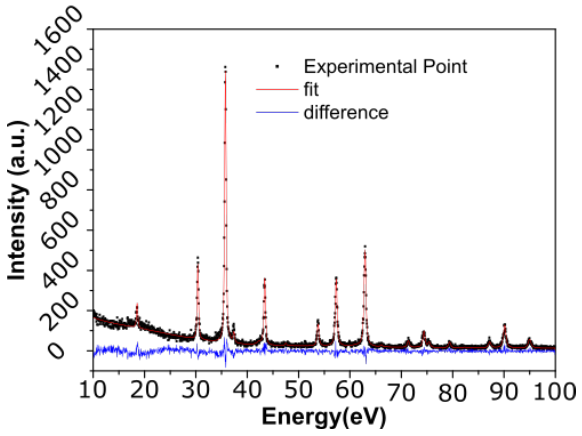

Figure S2. XRD measurement of synthetized VIP3 particles. The presented

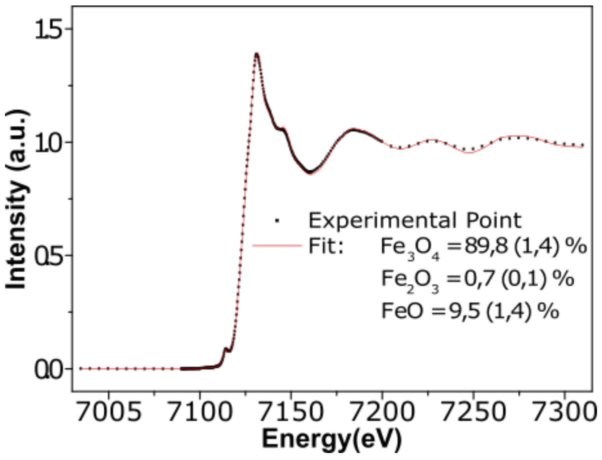

Figure S3. XANES measurement of VIP3 particles.

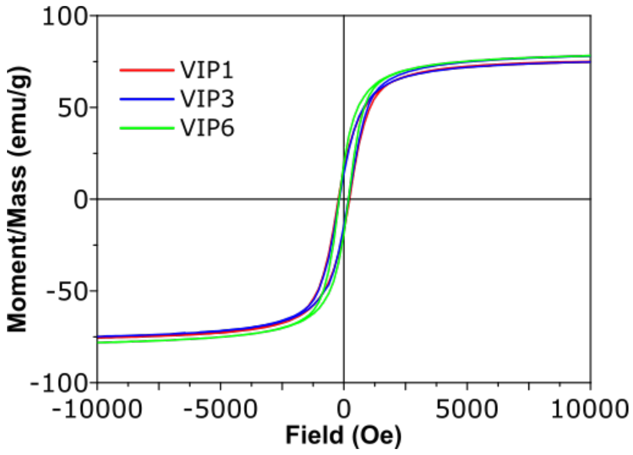

Figure S4. Magnetization curve measured for VIP1 (red), VIP3 (blue) and VIP6 (green).

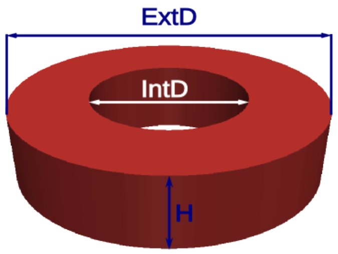

Figure S5. Representative image of a simulated nanoparticle used for the phase diagram composition.

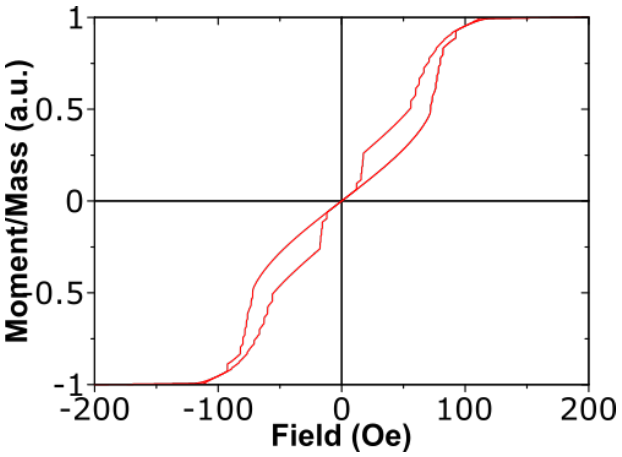

Figure S6. Simulated magnetic hysteresis for a monodispersive ensemble of randomly oriented nanorings.

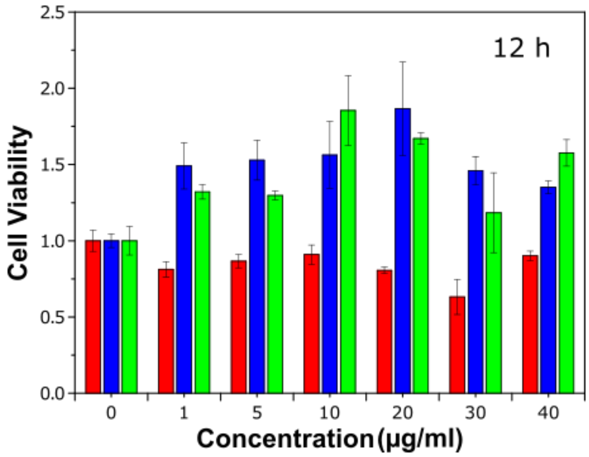

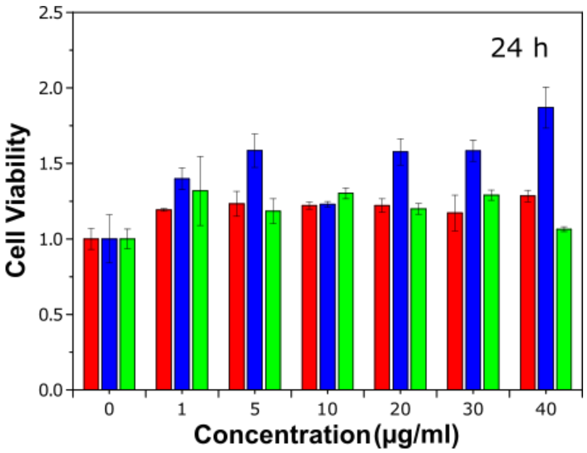

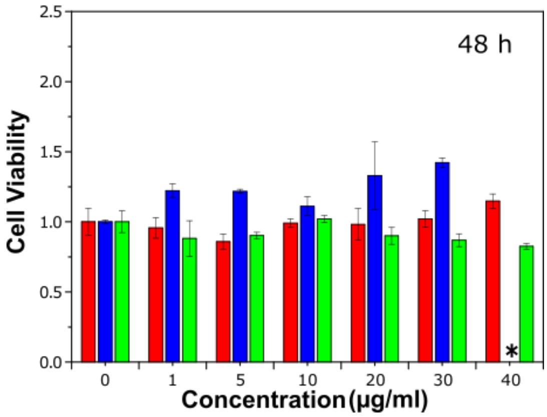

Figure S7. Cytotoxicity tests for 12, 24 and 48 hours of nanoparticle incubation determined by MTT assay. VIP1 red; VIP3 blue; VIP6 green

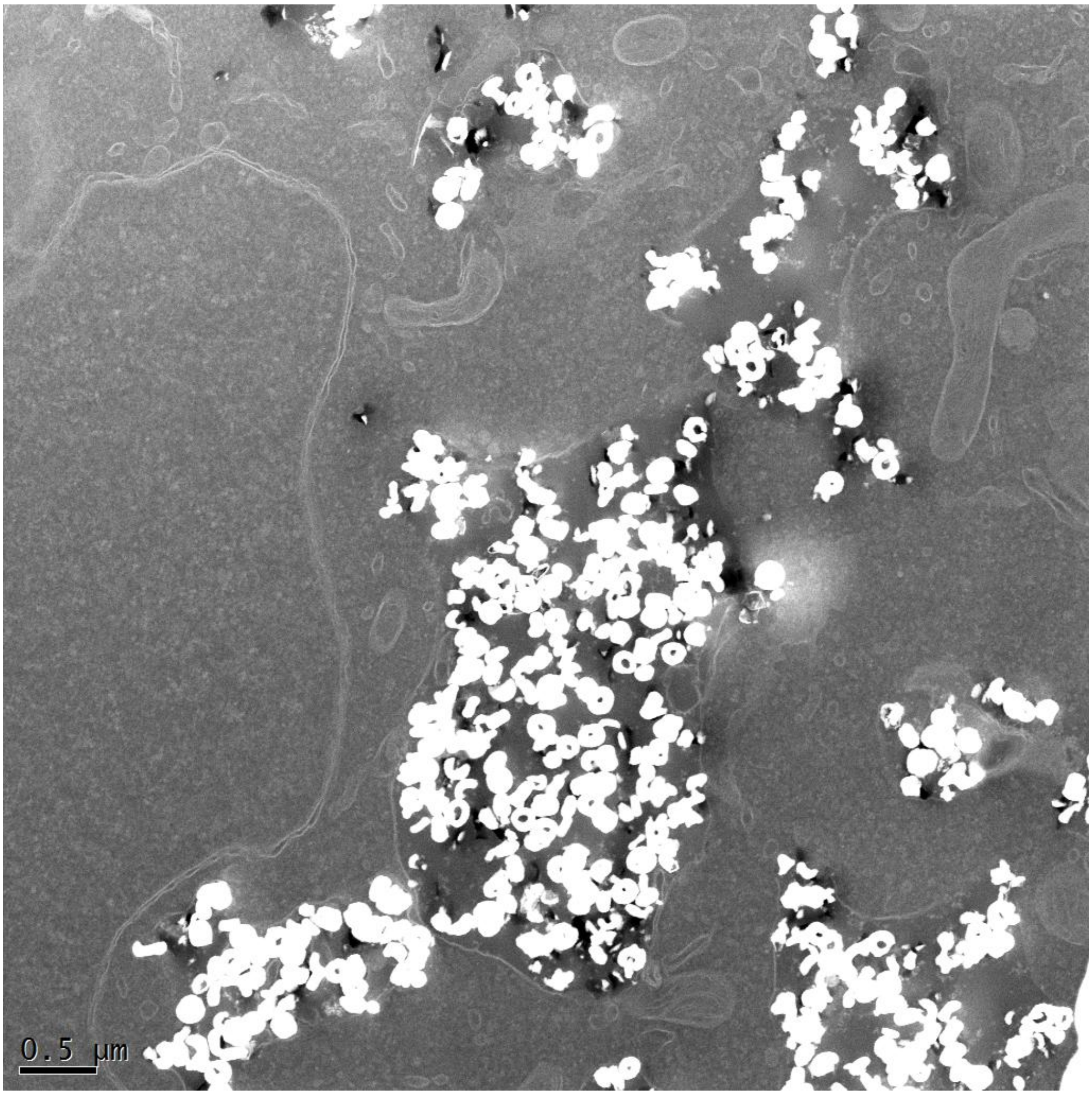

Figure S8. STEM ADF imaging for detailing cell.

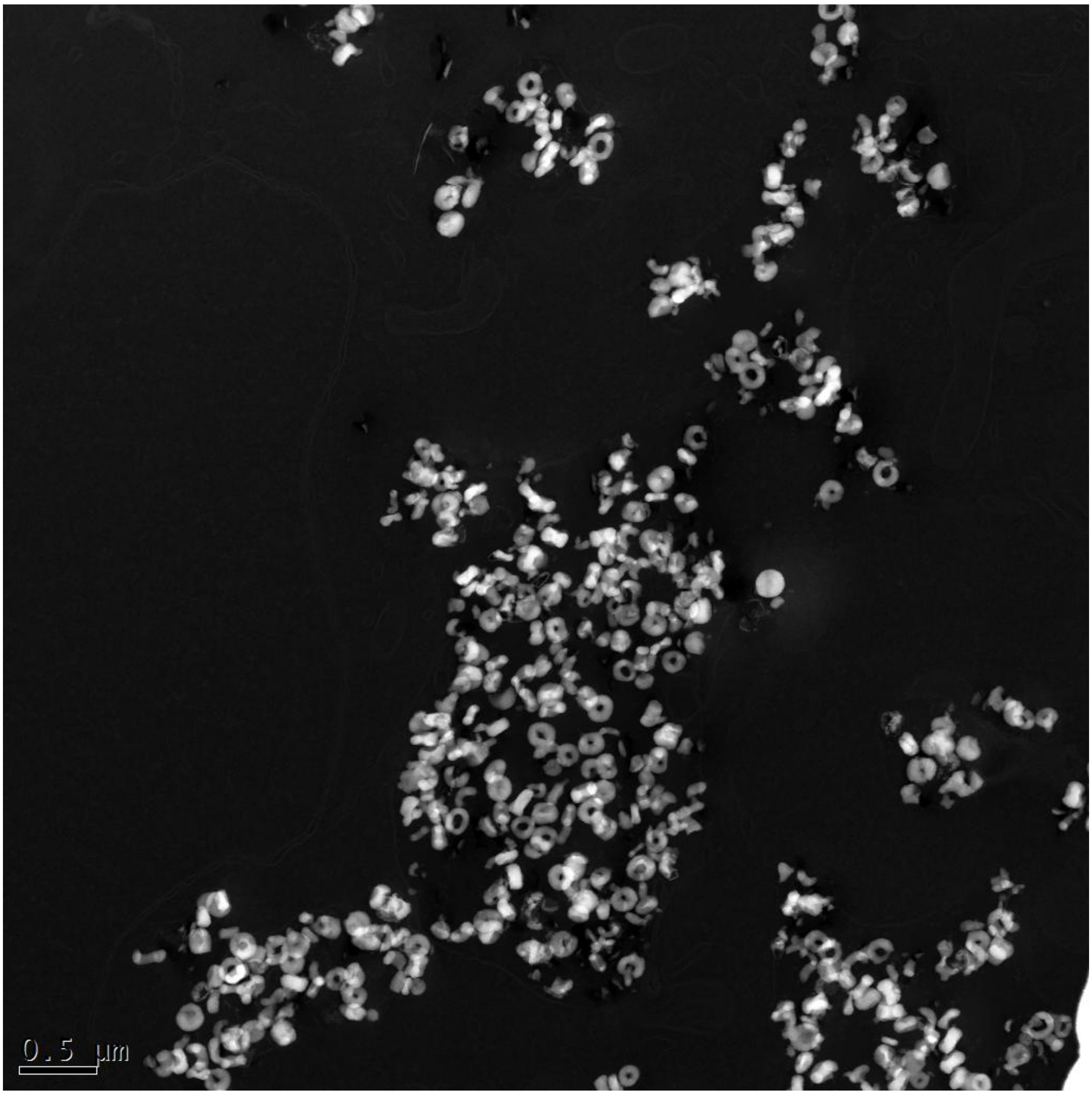

Figure S9. STEM HAADF for detailing VIP1 structure with distinctive ring-shape structure.

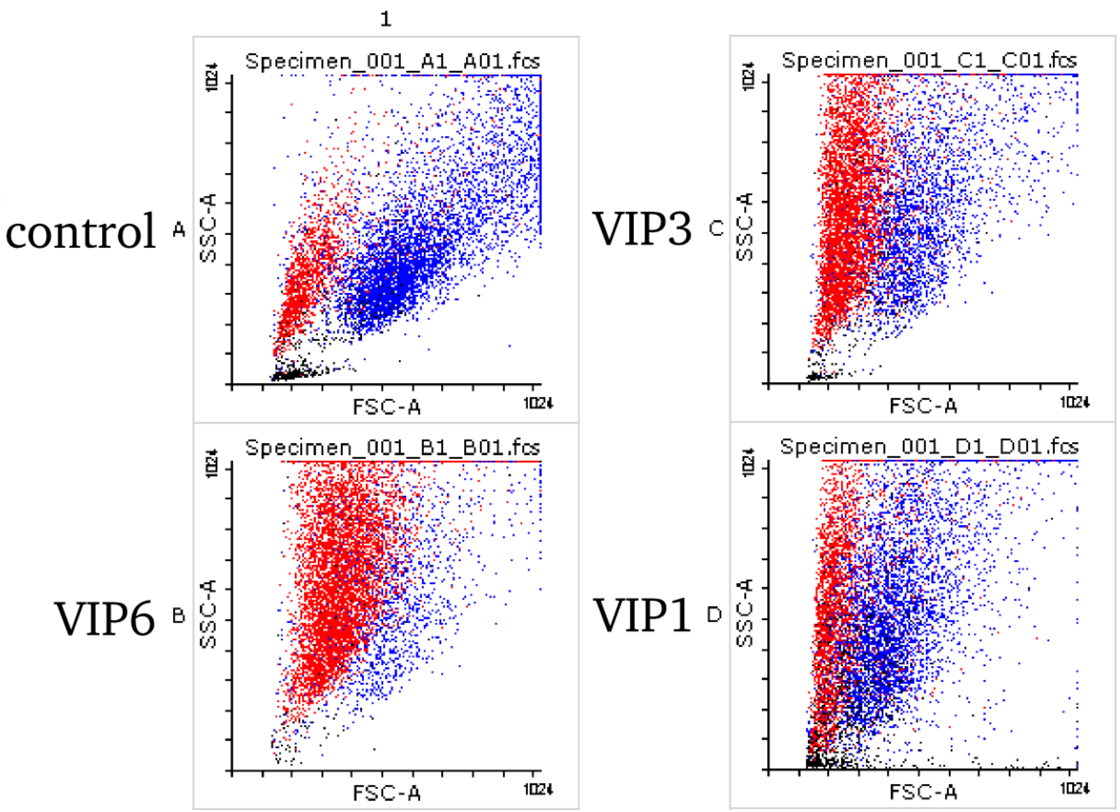

Figure S10. Flow Cytometry measurements: Side Scattering signal (SSC) vs Forward Scattering signal (FSC). All red points refer to dead cells and blue to live cells.
